# A novel immune-related long non-coding RNA signature improves the prognosis prediction in the context of head and neck squamous cell carcinoma

**DOI:** 10.1080/21655979.2021.1943284

**Published:** 2021-06-24

**Authors:** Lin Chen, Zhimou Cai, Kexing Lyu, Zhiwei Cai, Wenbin Lei

**Affiliations:** a Department of Otolaryngology, The First Affiliated Hospital of Sun Yat-sen University, Guangzhou, Guangdong, P.R. China; b Guangzhou Brain Hospital, Guangzhou Medical University, Guangzhou, Guangdong, P.R. China

**Keywords:** Head and neck squamous cell carcinoma, long non-coding RNAs, tumor-infiltrating immune cells, prognosis, TCGA

## Abstract

The tumor immune microenvironment plays an important role in head and neck squamous cell carcinoma (HNSCC). Reliable prognostic signatures able to accurately predict the immune landscape and survival rate of HNSCC patients are crucial to ensure an individualized/effective treatment. Here, we used HNSCC transcriptomic and clinical data retrieved from The Cancer Genome Atlas and identified differentially expressed immune-related long non-coding RNAs (DEirlncRNAs). DEirlncRNA pairs were recognized using univariate analysis. Cox and Lasso regression analyses were used to determine the association between DEirlncRNA pairs and the patients’ overall survival and build the prediction model. Receiver operating characteristic curves and Kaplan–Meier survival curves were used to validate the prediction model. We then reevaluated the model based on the clinical factors, tumor-infiltrating immune cells, chemotherapeutic efficacy, and immunosuppression biomarkers. We built a risk score model based on 18 DEirlncRNA pairs, closely related to the overall survival of patients (hazard ratio: 1.376; 95% confidence interval: 1.302–1.453; *P* < 0.0001). Compared with two recently published lncRNA signatures, our DEirlncRNA pair signature had a higher area under the curve, indicating better prognostic performance. Additionally, the signature score positively correlated with aggressive HNSCC outcomes (low immunity score, significantly reduced CD8 + T cell infiltration, and low expression of immunosuppression biomarkers). However, high-risk patients might have high chemosensitivity. Overall, the lncRNAs signature established here shows promising clinical prediction and the effective disclosure of the tumor immune microenvironment in HNSCC patients; therefore, such signature might help distinguish patients that could benefit from immunotherapy.

## Graphical abstract

**Figure:**
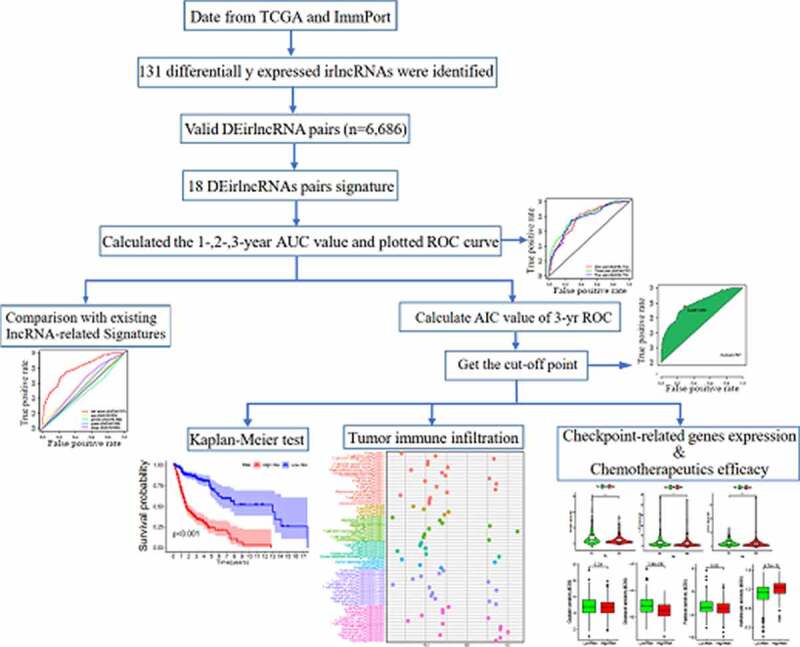

## Introduction

Head and neck tumors are the sixth most common type of cancer worldwide; head and neck squamous cell carcinoma (HNSCC) is the most common histological type [1]. Of note, HNSCC encompasses a heterogeneous group of tumors that arise in the oral cavity, larynx, and pharynx, mainly associated with tobacco and alcohol consumption, and human papillomavirus infection [2–4]. However, because the early symptoms of HNSCC are not obvious, the 5-year survival rate is below 50%. Furthermore, HNSCC is characterized by a high lymph node metastasis rate, and local recurrence and metastasis can reduce the survival rate to 35% [5]. Currently, HNSCC is estimated to have a global annual incidence and mortality rate of 900000 cases and 450000 deaths, respectively [1].

HNSCC is a disease characterized by profound immunosuppression [6]. Thus, immune checkpoint inhibitor (ICI) therapy is a new hope for HNSCC patients. In fact, recently, considerable progress has been made in ICI-based HNSCC treatment. However, the response rate of recurrent or metastatic HNSCC to PD-1/PD-L1 inhibitors is still low, in the range of 13.3–22% as per previous clinical trials [6]. Therefore, the identification of biomarkers that can effectively predict the efficacy of ICIs is crucial for patient selection. Generally, the expression of PD-L1 is used as the representative marker to predict the efficacy of ICIs. However, for most tumors, the detection of PD-L1 expression alone is not sufficient [7–9].

Long non-coding RNAs (lncRNAs) are functional RNA molecules with a length of more than 200 nucleotides that have minimal or no protein-encoding role [10]. It is increasingly recognized that lncRNAs can interact with DNA, RNA, and the proteins that regulate gene expression at the transcriptional and post-transcriptional levels. They are also associated with various types of cancer [10,11]. Recently, several lncRNA signatures have been established for prognostic prediction in some cancers, including HNSCC. For instance, Mao et al. [12] identified an eight-lncRNA signature that is an independent prognostic factor in HNSCC patients. Additionally, Bao et al. [13] identified genome instability-associated lncRNAs and constructed a signature to evaluate the prognosis of breast cancer patients.

The diagnosis, evaluation, and treatment of cancer are closely related to the tumor immune microenvironment, especially to the infiltrating immune cells [14–16]. Recent research has confirmed that lncRNAs are critical regulators of gene expression in the immune system; lncRNAs direct the expression of genes related to the development/activation of diverse immune cells, impacting tumor immune cell infiltration and, consequently, the immune microenvironment [17,18]. Therefore, we hypothesized that immune-related lncRNAs (irlncRNAs) influence immune cell infiltration in HNSCC, and, therefore, the treatment response and prognosis. In this study, we aimed to identify irlncRNAs to i) investigate the relationship between irlncRNAs and immune cell infiltration, ii) construct a powerful prognostic model in order to assess the risk in HNSCC, and iii) predict the response of HNSCC patients to immunotherapy.

## Materials and methods

### Data collection

HNSCC RNAseq expression profile data were downloaded from The Cancer Genome Atlas (TCGA) database (https://portal.gdc.cancer.gov/); tumor and normal tissue sequencing information from 501 patients with HNSCC and 44 healthy individuals were retrieved, together with the clinical and follow-up data in the context of the HNSCC patients. Valid data were extracted after the elimination of data from patients with a follow-up time of no more than 30 days and of duplicated data. ‘Ensembl_Stable_id’ was converted to ‘Gene Symbol’ in the RNAseq expression profile according to the GTF file downloaded from Ensembl (http://asia.ensembl.org) and annotated to distinguish the mRNAs and lncRNAs for subsequent analysis. A list of immune-related genes (ir-genes) was downloaded from the ImmPort database (http://www.immport.org), and correlation analyses were conducted between the ir-genes and all lncRNAs to screen for irlncRNAs. Immune gene correlation coefficients higher than 0.4 or lower than −0.4 and with a *P* value lower than 0.001 were considered as indicative of irlncRNAs. The differentially expressed irlncRNAs (DEirlncRNAs) were identified through the ‘limma’ R package; irlncRNAs with a false discovery rate < 0.05 and |log_2_FC| > 1.5 were defined as DEirlncRNAs.

### DEirlncRNAs pairing

We used a novel modeling algorithm (with pairing and iteration), to construct a two-biomarker combinations-based irlncRNA signature to improve the accuracy of the cancer prognostic model. The DEirlncRNAs were cyclically singly paired, and a 0-or-1 matrix was constructed. If the expression of the former lncRNA was higher than that of the latter, the matrix was defined as 1; otherwise, it was defined as 0. The constructed 0-or-1 matrix was further screened. A valid match was defined when the number of DEirlncRNA pairs, of which the expression was set as 0 or 1, accounted for more than 20% or less than 80% of the total number of pairs.

### Building the prognostic DEirlncRNA pair-based signature

Univariate Cox analysis was performed using the ‘survival’ R package to screen DEirlncRNA pairs of prognostic value. DEirlncRNA pairs with *P* < 0.01 were considered significantly related to the prognosis of HNSCC. Next, Lasso regression analysis, performed using the ‘glmnet’ and ‘survival’ R packages, was used to select the minimum error value to eliminate overfitting of the data. The optimal penalty parameter ‘lambda’ value was calculated via 1000 cross-validations [19]. Then, multivariate analyses were performed using the Cox regression model to identify the prognostic DEirlncRNA pairs in HNSCC. Based on the coefficients from the multivariate regression analysis and the status of the DEirlncRNA pairs, we constructed a DEirlncRNA pair signature (DEirLnc-PSig) for the prediction of the clinical outcome, as follows:

$$DEirLnc-PSig\left(patient\right)=\overset{n}{\underset{i=1}{\sum }}\beta i*Pi$$

where βi indicates the coefficient for each DEirlncRNA pair and Pi indicates the DEirlncRNA pair status on the 0-or-1 matrix.

### Validation of the constructed risk-assessment model

A time-dependent receiver operating characteristic (ROC) curve and the respective area under the curve (AUC) were obtained using the ‘survivalROC’ R package to predict the prognosis accuracy of DEirLnc-PSig in patients with HNSCC. The 1-, 2-, and 3-year ROC curves and the AUCs of the models were plotted. The Akaike information criterion (AIC) values for each data point of the 3-year ROC curve were also estimated to identify the maximum inflection point, considered as the cutoff value for the separation of patients into the high-risk and low-risk groups, with high or low DEirLnc-PSig expression, respectively [20].

The Kaplan–Meier method was used to construct survival curves, and the log-rank test was used to assess the difference in the survival between the high- and low-risk groups with a significance level of 0.05. The R packages used in these steps were ‘survival’ and ‘survminer’. To verify the clinical application of DEirLnc-PSig, multivariate Cox regression and stratified analyses were further used, to assess the independence of DEirLnc-PSig from other key clinical factors. A forest map was drawn to demonstrate the results using again the R packages ‘survival’ and ‘survminer’. In addition, we compared our defined DEirLnc-PSig with two previously published lncRNA signatures to further assess the performance of our prognostic model.

### RNA preparation, cDNA synthesis, and qRT-PCR validation

Total RNA from frozen tissue specimens was extracted using the TRIzol reagent (Invitrogen, Carlsbad, CA, USA) according to the manufacturer’s protocol. The RNA quantity and quality were determined using a NanoDrop 2000 spectrophotometer (Thermo Fisher Scientific, Waltham, MA, USA). Reverse transcription was conducted using the Prime Script RT Master Mix Kit (TAKARA BIO INC, Japan) according to the manufacturer’s protocol. qRT-PCR was performed using the SYBR Green Premix Pro Taq HS qPCR Kit (Agbio, Hunan, China) on the LightCycle480 II system (Roche Applied Science, Basel, Switzerland). The relative quantification of each gene was achieved after normalization to the expression levels of *GAPDH* using the comparative CT method. The mean Ct value of each gene minus the mean Ct value of *GAPDH* was defined as the ∆ Ct. The primer sets used in this study are listed in Table S1. Since, as mentioned above, the risk model in this study was constructed based on two-biomarker combinations, we evaluated patient risk via the comparison of the ∆ Ct value of the matched genes.

### Investigation of tumor-infiltrating immune cells

To explore the differences in immune cell infiltration between the different risk groups, we used the xCell [21], TIMER [22], QUANTISEQ [23], MCPcounter [24], EPIC [25], CIBERSORT-abs [26], and CIBERSORT [27] methods in the context of the HNSCC dataset (TCGA project). Differences in the numbers of distinct immune-infiltrating cell types between the high- and low-risk groups in the constructed model were analyzed using the Wilcoxon’s signed-rank test. The correlation between the risk scores and immune cell infiltration was evaluated using Spearman’s correlation analysis. The significance threshold was set as *P* < 0.05. The ‘limma’, ‘scales’, ‘ggplot2’, ‘ggtext’, and ‘ggpubr’ R packages were used for the above operations.

### Exploration of the significance of the model for clinical treatment

Using the ‘pRRophetic’ R package, we calculated the half inhibitory concentration (IC_50_) of the commonly administered chemotherapeutic drugs in the HNSCC dataset (TCGA project) to evaluate the utility of the model for the clinical treatment of HNSCC. Anti-tumor drugs such as cisplatin, docetaxel, methotrexate, and paclitaxel are recommended for the treatment of HNSCC as per the National Comprehensive Cancer Network guidelines [28]. The difference in the IC_50_ between the high- and low-risk groups was compared using the Wilcoxon’s signed-rank test.

### Analysis of the expression of immunosuppression biomarkers in the context of ICIs

The relationship between the constructed model and the expression of genes related to ICIs was analyzed using the ‘limma’ and ‘ggpubr’ R packages and visualized in the form of violin plots.

## Results

LncRNAs are important factors for the prognosis of HNSCC patients. We hypothesized that immune-related lncRNAs (irlncRNAs) influence immune cell infiltration, treatment response, and prognosis in the context of HNSCC patients. In this study, we aimed to investigate the relationship between irlncRNAs and immune cell infiltration, and to construct a powerful prognostic model to assess HNSCC risk and predict the response of patients to immunotherapy. We identified DEirlncRNAs using an HNSCC cohort retrieved from TCGA. DEirlncRNA pairs were recognized using univariate analysis. Cox and Lasso regression analyses were also used to identify potential DEirlncRNA pairs related to the OS and build the prediction model. Overall, the prediction model established here reflects the HNSCC tumor immune microenvironment and can be used to predict HNSCC sensitivity to different treatment agents.

### Identification of irlncRNAs and DEirlncRNAs

**Figure 1.**
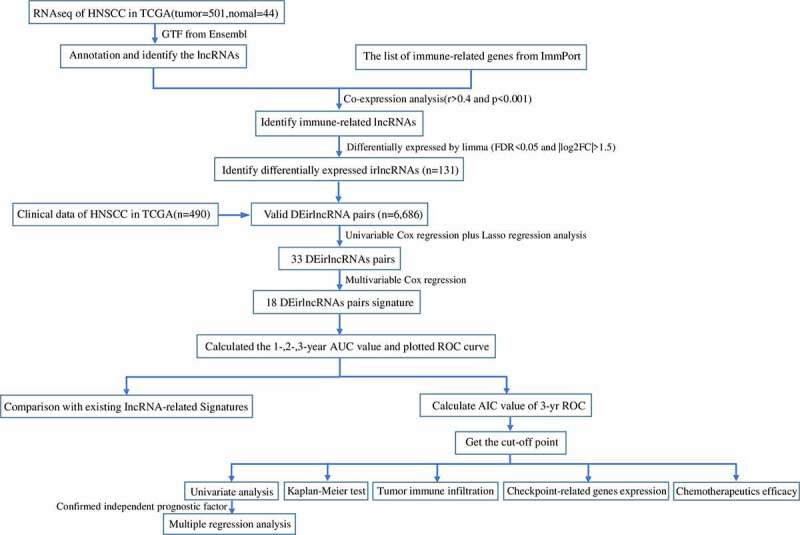
Flow chart of this study

Figure 1 presents the study design. A total of 805 irlncRNAs were identified after the co-expression analysis of ir-genes and lncRNAs. Differential expression analysis was subsequently performed using the ‘limma’ R package, and 132 DEirlncRNAs (116 upregulated and 16 downregulated) were identified between the tumor and normal tissues (Figure S1).

### Establishment of the DEirlncRNA pairs and DEirlncRNA-pair signature

**Figure 2.**
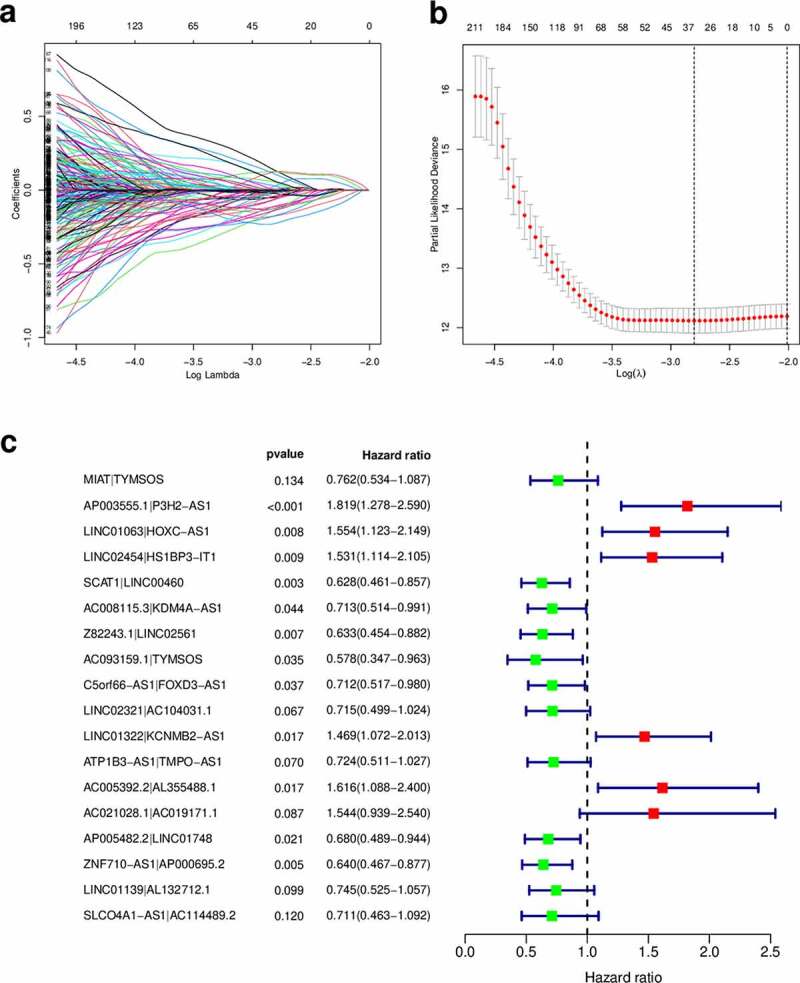
Diagrams of the lasso and multivariable Cox regression analyses. (a) Lasso regression analysis. (b) The penalty coefficient was used to minimize the mean square error of the models. (c) A forest graph of the 18 DEirlncRNA pairs identified via multivariable Cox regression analysis

**Figure 3.**
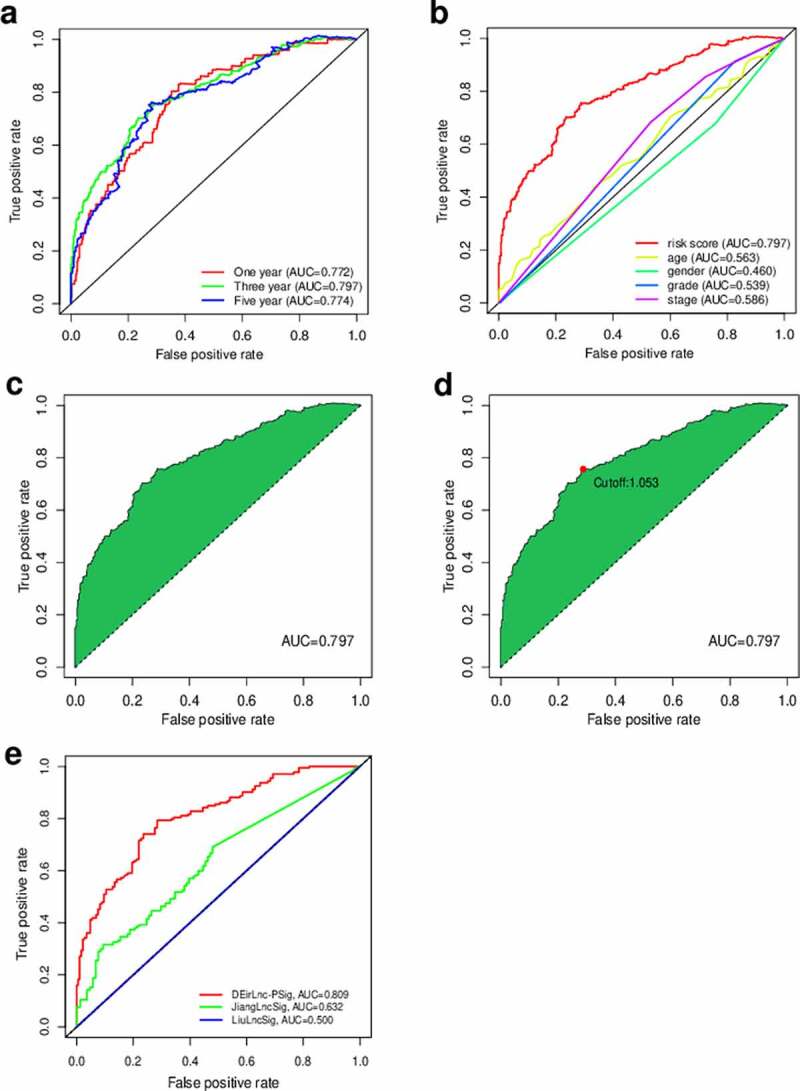
Prediction of the prognosis accuracy of DEirLnc-PSig in patients with HNSCC. (a) The 1-, 2-, and 3-year receiver operating characteristic (ROC) curves of the DEirLnc-PSig. (b) A comparison of the 3-year ROC curves with other common clinical characteristics showed the superiority of the risk score. (c, d) The maximum inflection point obtained using the Akaike information criterion. (e) ROC analysis with respect to the 3-year overall survival for DEirLnc-PSig, JiangLncSig, and LiuLncSig

Using an iteration loop and the 0-or-1 matrix screening based on the 132 DEirlncRNAs, 6686 valid DEirlncRNA pairs were identified. After univariate Cox regression analysis, 500 survival-related DEirlncRNA pairs were selected (*P* < 0.01). To further avoid data overfitting, Lasso regression analysis was employed to screen out 33 DEirlncRNA pairs that showed the highest correlation with survival (Figure 2(a,b)). These 33 DEirlncRNA pairs were further subjected to multivariate Cox regression analysis, and the 18 most significant DEirlncRNA pairs were identified and used for the development of the DEirlncRNA pair signature for HNSCC patients using the stepwise elimination method (Figure 2(c)). Of note, when we applied the 1-, 2-, and 3-year ROC curves to verify the predictive capacity of the DEirLnc-PSig the AUCs were almost all ~0.75, suggesting a satisfactory predictive performance (Figure 3(a)). Importantly, the predictive capacity of the DEirLnc-PSig was superior to that of other clinical features (Figure 3(b)).

### Clinical evaluation using the risk assessment model and analysis of the independence of the DEirLnc-PSig from other clinical factors

**Figure 4.**
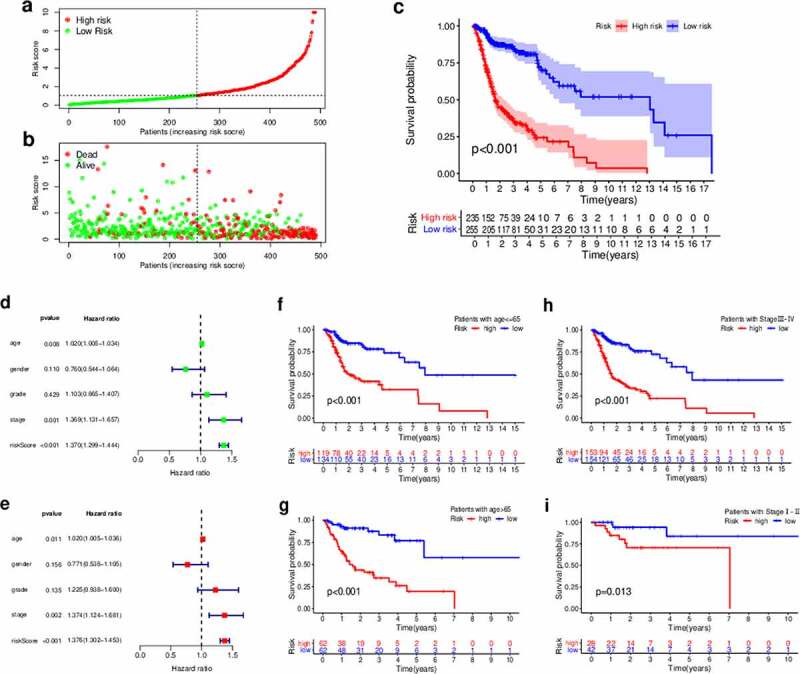
Prediction of the survival of HNSCC patients and identification of the risk factors using the risk score model and univariate and multivariate Cox analyses, respectively. (a, b) Patients were scored and grouped into a low-risk group (green) and a high-risk group (red). Then, scatter diagrams of the survival rate of patients were plotted based on the risk scores from low to high, with green indicating live patients and red indicating deaths. (c) Kaplan–Meier survival curves. (d) Univariate analysis. (e) Multivariate analysis. (f, g) Kaplan–Meier curve analysis of the overall survival (OS) in the high- and low-risk groups, in the context of young and old patients. (h, i) Kaplan–Meier curve analysis of the OS in the high- and low-risk groups in the context of early-stage and late-stage patients. Statistical analysis was performed using the log-rank test

We defined the maximum inflection point as the cutoff value in the 3-year ROC curve using the AUC values (Figure 3(c,d)). HNSCC patients (TCGA cohort) were then divided into the low-risk (n = 255) and high-risk (n = 235) groups. Interestingly, when we compared the survival in the two groups we found that the DEirLnc-PSig possessed a powerful ability to predict the differences in the prognosis of HNSCC. In fact, the OS was significantly better in the low-risk *versus* high-risk groups (*P* < 0.001, log-rank test; Figure 4(a–c).

Next, to evaluate whether the prognostic value of the DEirLnc-PSig was independent of the common clinical features, univariate and multivariate Cox regression analyses were performed using age, gender, pathologic grade, and clinical stage data, and our DEirLnc-PSig-based prognostic risk score model (Figure 4(d,e)). Importantly, the risk score model showed statistical differences, suggesting it can be used as an independent prognostic predictor. Of note, there were two other clinical factors, age, and clinical stage, defined as significant in the multivariate analysis. Therefore, a stratification analysis was performed to determine whether the DEirLnc-PSig possessed a prognostic value independent of the age and clinical stage. Patients (TCGA dataset) were stratified into the young (≤65 years, n = 124) and old (>65 years, n = 253) groups, followed by the subsequent categorization into the high- or low-risk groups using the DEirLnc-PSig. Importantly, there was a still significant difference in the OS between the high- and low-risk groups in both the young (*P* < 0.001, log-rank test) and old (*P* < 0.001, log-rank test; Figure 4(f, g) patient groups. Additionally, all HNSCC patients were stratified into the early- (stages I and II, n = 70) or late-stage (stages III and IV, n = 307) groups, followed by the same categorization into the high- or low-risk groups using the DEirLnc-PSig. Again, there was a significant difference in the OS between the high- and low-risk groups in both the early-stage and late-stage groups (*P* = 0.013 and *P* < 0.001, respectively, log-rank test; Figure 4(h,i)). Therefore, altogether, these results indicate that the DEirLnc-PSig is an independent prognostic factor associated with the OS of HNSCC patients.

### Comparison of the performances of the DEirLnc-PSig and other existing lncRNA-based signatures in the prediction of survival

We further compared the predictive performance of the DEirLnc-PSig with that of two recently published lncRNA signatures: the 3-lncRNA signature derived proposed by the study by Jiang *et al* (hereafter referred to as JiangLncSig) [29] and the 5-lncRNA signature proposed by Liu *et al* (hereafter referred to as LiuLncSig) [30] using the same TCGA patient cohort. As shown in Figure 3(e), the AUC of the 3-year ROC curve for the DEirLnc-PSig (AUC = 0.809) is significantly higher than those in the context of the JiangLncSig (AUC = 0.632) and LiuLncSig (AUC = 0.5). These results clearly highlight that the DEirLnc-PSig has better prognostic performance (with respect to the OS) than the two recently published lncRNA signatures.

### Validation of the gene signature using qRT-PCR

**Figure 5.**
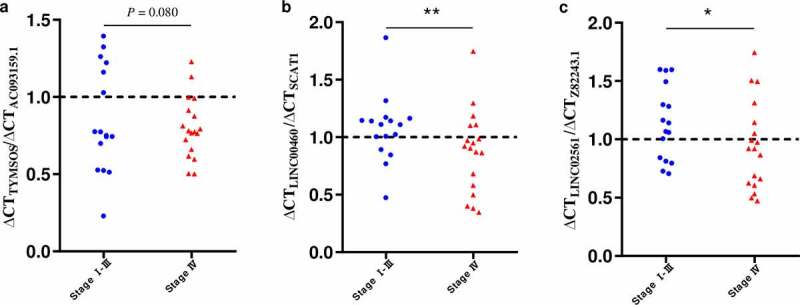
qRT-PCR analysis of the two-lncRNA combinations in HNSCC tissues at different developmental stages. (a) ∆ Ct _AC093159.1_/∆ Ct _TYMSOS_. (b) ∆ Ct _SCAT1_/∆ Ct _LINC00460_. (c) ∆ Ct _Z82243.1_/∆ Ct _LINC026561_. **P < 0.01, *P < 0.5

**Figure 6.**
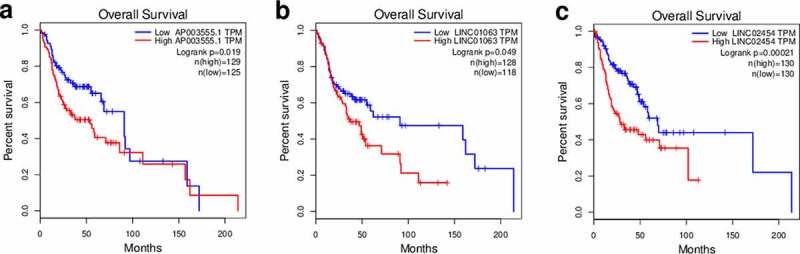
Overall survival analysis of HNSCC patients in the context of the expression of different risk factors. The GEPIA website was used. (a) *P3H2-AS1*, (b) *HOXC-AS1*, and (c) *HS1BP3-IT1.*

Next, using qRT-PCR, we evaluated the expression of the gene pairs comprising the top 5 coefficients in the context of the DEirLnc-Psig, in 34 HNSCC tissues. According to tumor tissue sources, the 34 HNSCC tissues were divided into two groups: stage I-III and stage IV. The qRT-PCR results showed that the ratio less than 1 of ∆ Ct _AC093159.1_/∆ Ct _TYMSOS_, ∆ Ct _SCAT1_/∆ Ct _LINC00460_ and ∆ Ct _Z82243.1_/∆ Ct _LINC02561_ (indicating the expression of the former lncRNA was higher than that of the latter), stage I-III group were significantly more than those in the stage Ⅳ group (*P* = 0.08, *P* < 0.01 and *P* < 0.05, respectively, chi-square test). Of note, since the Ct value is inversely proportional to the gene expression levels, in order to facilitate display, as shown in Figure 5(a–c), we used the reciprocal of the paired gene Ct value to represent the expression levels. On the other hand, for risk factors, such as AP003555.1/P3H2-AS1, LINC01063/HOXC-AS1 and LINC02454/HS1BP3-IT1, there were no statistical differences, as per the PCR results. Still, in order to further verify the accuracy of the model, gene expression profiling interaction analysis (GEPIA) (http://gepia.cancer-pku.cn/) was used performed in the context of survival [31]. Importantly, and as expected, a lower expression of *P3H2-AS1, HOXC-AS1*, and *HS1BP3-IT1* was associated with a longer OS (Figure 6(a–c); *P* = 0.019, *P* < 0.05 and *P* < 0.001, respectively).

### Estimation of tumor-infiltrating immune cells and the expression of immunosuppression biomarkers using the risk assessment model

**Figure 7.**
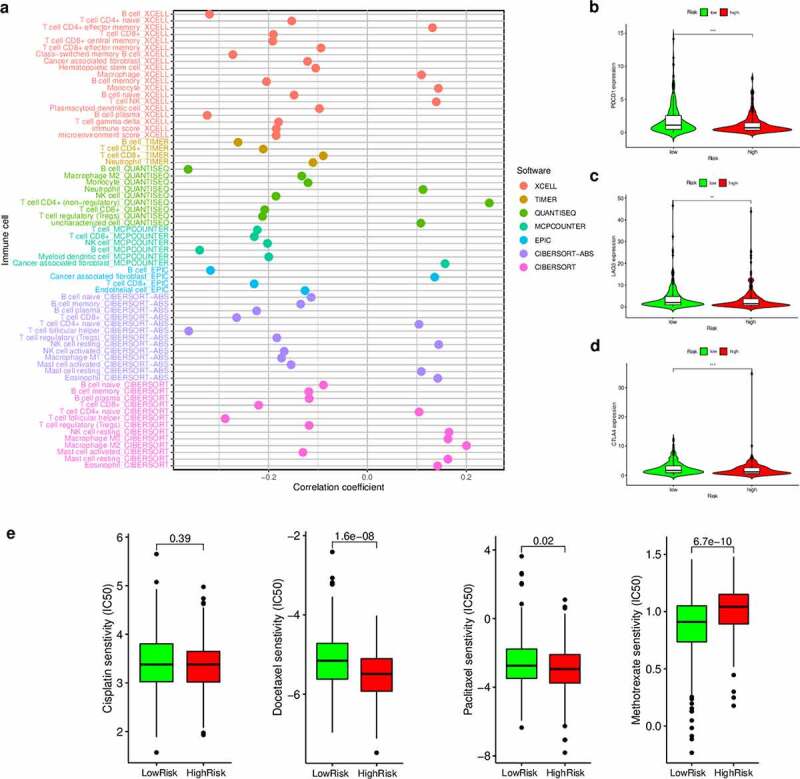
Estimation of tumor-infiltrating cells, immunosuppression molecules, and the IC_50_ values of chemotherapeutics using the risk assessment model. (a) Lollipop diagram of the correlation between the risk scores and immune cell infiltration. (b–d) Violin plots of the expression of genes related to Immune checkpoint inhibitors in the low- and high-risk groups. (e) Boxplots highlighting the difference in the IC_50_ values of cisplatin, docetaxel, methotrexate, and paclitaxel between the high- and low-risk groups

Since our model was based on irlncRNAs, we further investigated the potential relationship with the tumor immune microenvironment. Correlation analysis in the context of immune cells infiltration revealed that the high-risk group was positively associated with CD4 + T cells, cancer-associated fibroblasts, monocytes, and macrophages, and negatively associated with CD8 + T cells and regulatory T cells, as revealed by the Wilcoxon signed-rank test (Table S2 and Figure S2). In addition, the analysis using XCELL revealed that the immune scores in the low-risk group were significantly higher than those in the high-risk group (*P* = 0.0038); these results suggest that the tumors in the low-risk group had more immune cell infiltration than those in the high-risk group. A detailed Spearman’s correlation analysis was further conducted, and the resulting diagram exhibited a lollipop shape, as shown in Figure 7(a). Since immunotherapy with ICIs has dramatically changed the HNSCC treatment panorama, improving the survival of HNSCC patients, next, we investigated whether the risk assessment model was related to the expression of ICI-related biomarkers. Curiously, we discovered that the high-risk scores were positively correlated with a low expression of *PDCD1* (*P* < 0.001, Figure 7(b)), *LAG3* (*P* < 0.01, Figure 7(c)), and *CTLA4* (*P* < 0.001, Figure 7(d)).

### Correlation between the risk assessment model and chemosensitivity

As chemotherapy is commonly used to treat HNSCC, we also attempted to identify associations between the risk scores and the efficacy of different chemotherapy agents in the treatment of HNSCC using the HNSCC dataset (TCGA project). Importantly, we found that a high-risk score was associated with a lower IC_50_ value for chemotherapeutic drugs, such as docetaxel (*P* < 0.001) and paclitaxel (*P* = 0.02), but with a higher IC_50_ value for methotrexate (*P* < 0.001) (Figure 7(e)). Altogether, these results indicate that our model also acts as a potential predictor of chemosensitivity.

## Discussion

In recent years, many studies have highlighted lncRNAs as important factors for the prognosis of HNSCC patients [32]. In fact, many prognostic lncRNA expression signatures have been applied to both the diagnosis and prognosis prediction in the context of HNSCC. However, most signatures are based on the quantification of the expression levels. Here, we attempted to construct a reasonable model based on two-lncRNA combinations, instead of on their specific expression levels. Only pairs with high or low expression were detected (*versus* the examination of the specific expression levels of each lncRNA), aiming to increase the diagnosis/prognosis accuracy in the context of cancer, thinking on clinical practicability. Our findings showed that this novel model has a good predictive performance, better than those of two previously published expression-based lncRNA signatures. Of note, the risk score derived from the DEirLnc-PSig is reliable and independently predicts the prognosis in the context of HNSCC.

Some of the irlncRNAs previously associated with the malignant phenotypes of various cancers, including MIAT [33], TYMSOS [34], LINC01063 [35], HOXC-AS1 [36], LINC02454 [37], SCAT1 [38], and LINC01322 [39] were identified in the process of modeling in this study. Furthermore, previous studies have also shown that LINC00460 [40], C5orf66-AS1 [41], and TMPO-AS1 [42], also identified in our study, are associated with the prognosis and treatment outcomes of HNSCC. In fact, Jiang et al. [40] revealed that LINC00460 promotes epithelial-mesenchymal-transition in HNSCC via the facilitation of the transfer of peroxiredoxin-1 into the nucleus. Additionally, Lu et al. [41] first reported that C5orf66-AS1 is able to prevent the progression of oral squamous cell carcinoma, inhibiting tumor cell growth and metastasis via the regulation of CYC1 expression. Last but not least, Xing et al. [42] identified the novel TMPO-AS1/miR-320a/SOX4 pathway associated with nasopharyngeal carcinoma progression; interestingly, these authors suggested that TMPO-AS1 may be a potential therapeutic target for nasopharyngeal carcinoma. However, some lncRNAs were identified for the first time in the model established in the present study, suggesting that the proposed model could identify novel biomarkers for further research. Importantly, we validated these biomarkers using qRT-PCR and found that the gene expression rate of protective factors such as AC093159.1/ TYMSOS, SCAT1 /LINC00460 and Z82243.1/LINC02561 was significantly higher in early versus late tumor stages. Additionally, although no obvious differences in the expression of risk factors were found in the context of different tumor stages, survival analysis showed that such risk factors were closely related to survival. Therefore, overall, these results further validate the model in this study.

It should be noted that HNSCC is a type of immunosuppressive disease [43]. Despite the effectiveness of targeted therapies and immunotherapy agents in the context of HNSCC, not all patients respond to such treatments and the development of resistance is almost universal after a period of time. In fact, in clinical practice, ICI therapy does not appear to improve the survival of HNSCC patients. Instead, precision medicine approaches that seek to individualize therapy through the use of predictive biomarkers and stratification strategies have been the key to improve the therapeutic outcomes of HNSCC patients [32,43]. Hence, it is essential to develop sensitive predictive biomarkers, essential for the deeper understanding of the heterogeneity and complexity of the tumor immune microenvironment; only through this approach can we finally improve the efficacy of tumor immunotherapy. For instance, an ideal signature would not only predict the prognosis of cancer patients, but also reflect the characteristics of the tumor and its immune microenvironment, both directly associated with disease progression and treatment response. Therefore, here we established a prediction model using irlncRNAs and evaluated the relationships between the risk scores drawn from the DEirLnc-Psig and immune checkpoints, the abundance of tumor-infiltrating immune cells, and response to chemotherapy.

Tumor-infiltrating immune cells, part of the tumor immune microenvironment, are recognized as highly important for prognosis prediction; in fact, the responsiveness to immune checkpoint blockade may be related to the features of the tumor immune microenvironment. Therefore, here, to explore the relationship between risk scores and tumor-infiltrating immune cells, we used seven commonly acceptable methods to estimate the abundance of infiltrating immune cells, including XCELL, TIMER, QUANTISEQ, MCPcounter, EPIC, CIBERSORT-abs, and CIBERSORT. Multiple studies have indicated that the greater infiltration of CD8 + T cells and high immune scores are correlated with better response to standard chemotherapy and immune checkpoint blockade therapy in cancer patients [44,45]. Our analyses revealed that the high-risk group, as per the DEirLnc-PSig was negatively associated with CD8 + T cell infiltration and immune scores. Correspondingly, the high-risk group was also negatively correlated with immune checkpoint-related biomarkers, such as *PDCD1, LAG3*, and *CTLA4*. Therefore, our results suggest that patients in the high-risk group identified by our model might not benefit from immunotherapy. However, this conclusion needs to be verified through further studies, especially in the context of clinical trials. On the contrary, the estimated IC_50_ of cisplatin, paclitaxel, doxorubicin, and methotrexate indicated that patients in the high-risk group could be more sensitive to chemotherapy than those in the low-risk group. Thus, altogether, our results suggest that this model may be particularly helpful to identify patients at a high clinical risk, the ones that will benefit from chemotherapy *in lieu* of immunotherapy. However, further benchwork and clinical studies are needed for further validation.

## Conclusion

In summary, our study reveals a comprehensive landscape of the tumor immune microenvironment in HNSCC and provides a promising prognostic tool in the context of HNSCC. For instance, patients in the high-risk group, as per the DEirLnc-PSig, negatively associated with CD8 + T cell infiltration and immune scores, have a worse prognosis. On the contrary, the low-risk group whose immune score was significantly higher than that of the high-risk group, was associated with a better prognosis. Therefore, overall, these details suggest that patients with low-risk scores may benefit from ICI therapy, while those with high-risk scores should receive chemotherapy rather than ICI therapy. However, prospective studies are needed to verify the prognosis accuracy of the established model and test its clinical utility.

## Supplementary Material

Supplemental Material

## Data Availability

The data that support the findings of this study are openly available in TCGA repository at https://portal.gdc.cancer.gov/.

## Research Highlights

- A DEirlncRNA-pair signature was identified to predict the OS of HNSCC patients.
- Different tumour-infiltrating immune cells are enriched in different risk groups.
- This tool may help distinguish HNSCC patients that could benefit from immunotherapy.
