# Supplementary material for: A novel immune-related long non-coding RNA signature improves the prognosis prediction in the context of head and neck squamous cell carcinoma
Source: Bioengineered. 2021 Jun 24;12(1):2311–25. doi: 10.1080/21655979.2021.1943284 (PMC8806432; doi:10.1080/21655979.2021.1943284)
Supplement: Supplemental Material [file KBIE_A_1943284_SM5115.docx]

**Table S1. DNA Oligos of primers**

| **Gene** | **primer sequence** | |
| --- | --- | --- |
| **AC093159.1** | **Forward primer** | CCACAGAGGGGTCAGGGAAGTATC |
|  | **reverse primer** | CTAGGCAGCAGGCAAGGTGAAC |
|  |  |  |
| **TYMSOS** | **Forward primer** | GGCGTCACCTCTCAGGCTGTAG |
|  | **reverse primer** | GAAATCCTGGGGCAGATCCAACAC |
|  |  |  |
| **SCAT1** | **Forward primer** | TACCCTCCTGAGTGCTGACCAAC |
|  | **reverse primer** | TTCTCTTCTCCTCCCTCGGTTCTTC |
|  |  |  |
| **LINC00460** | **Forward primer** | GCACACTTCTCGGCTAAGAGTCAC |
|  | **reverse primer** | GTCGTAACCTTCGTTCTCATCCACTG |
|  |  |  |
| **Z82243.1** | **Forward primer** | GAGAGGCAGCATAGAGGGTAGAGG |
|  | **reverse primer** | TGGAGATGAGGAGACACAGAAGTATCC |
|  |  |  |
| **LINC02561** | **Forward primer** | ATCAAGCAGTGGACCAACCATTAGC |
|  | **reverse primer** | GCAGCCCTGAGTTTCTACAGTCTTTC |
|  |  |  |
| **AP003555.1** | **Forward primer** | GCGAACGATGACTACGAATGAGAGG |
|  | **reverse primer** | ACCCAGAAACAAGCCCGAAACG |
|  |  |  |
| **P3H2-AS1** | **Forward primer** | TGCCTTAACTGATGACATTCCACCAC |
|  | **reverse primer** | AGCCAGGATGAGCCAGGAGAAG |
|  |  |  |
| **LINC01063** | **Forward primer** | TCTCCTTCCTGTGCCGAGTGTG |
|  | **reverse primer** | GAGTCGCTCTAGCCAATCACCTTC |
|  |  |  |
| **HOXC-AS1** | **Forward primer** | GACCGAGCTTGAAGAAGTGTAGGAG |
|  | **reverse primer** | GGAAGTGTCGCAGAGATGGAGTTG |
|  |  |  |
| **LINC02454** | **Forward primer** | AACATCGTCCTCCTCACCTCCTG |
|  | **reverse primer** | GTCTCCATGCACAGCCACTTCC |
|  |  |  |
| **HS1BP3-IT1** | **Forward primer** | ACATGCTCTCCTGGGCTTACTCC |
|  | **reverse primer** | GACGCCTCCATCTGTGCTTGTG |

**Table S2. The detail comparison results of correlation ship between tumour infiltrating immune** **cells and risk sore.**

| immune cell_method | cor | pvalue |
| --- | --- | --- |
| B cell memory_CIBERSORT | -0.11854 | 0.008624 |
| B cell memory_CIBERSORT-ABS | -0.13494 | 0.002763 |
| B cell memory_XCELL | -0.20416 | 5.22E-06 |
| B cell naive_CIBERSORT | -0.08914 | 0.048608 |
| B cell naive_CIBERSORT-ABS | -0.11382 | 0.011693 |
| B cell naive_XCELL | -0.14831 | 0.000992 |
| B cell plasma_CIBERSORT | -0.11778 | 0.009066 |
| B cell plasma_CIBERSORT-ABS | -0.22396 | 5.48E-07 |
| B cell plasma_XCELL | -0.32395 | 1.96E-13 |
| B cell_EPIC | -0.31728 | 6.38E-13 |
| B cell_MCPCOUNTER | -0.33911 | 1.19E-14 |
| B cell_QUANTISEQ | -0.36241 | 1.18E-16 |
| B cell_TIMER | -0.26153 | 4.18E-09 |
| B cell_XCELL | -0.31926 | 4.51E-13 |
| Cancer associated fibroblast_EPIC | 0.136037 | 0.002547 |
| Cancer associated fibroblast_MCPCOUNTER | 0.156959 | 0.000488 |
| Cancer associated fibroblast_XCELL | -0.12111 | 0.007274 |
| Class-switched memory B cell_XCELL | -0.27205 | 9.21E-10 |
| Endothelial cell_EPIC | -0.12614 | 0.005169 |
| Eosinophil_CIBERSORT | 0.141797 | 0.001651 |
| Eosinophil_CIBERSORT-ABS | 0.142203 | 0.001601 |
| Hematopoietic stem cell_XCELL | -0.10463 | 0.020533 |
| immune score_XCELL | -0.18419 | 4.10E-05 |
| Macrophage M0_CIBERSORT | 0.162777 | 0.000297 |
| Macrophage M1_CIBERSORT-ABS | -0.17361 | 0.000112 |
| Macrophage M2_CIBERSORT | 0.20021 | 7.98E-06 |
| Macrophage M2_QUANTISEQ | -0.13274 | 0.003241 |
| Macrophage_XCELL | 0.109264 | 0.015532 |
| Mast cell activated_CIBERSORT | -0.13045 | 0.00382 |
| Mast cell activated_CIBERSORT-ABS | -0.15451 | 0.000599 |
| Mast cell resting_CIBERSORT | 0.162595 | 0.000301 |
| Mast cell resting_CIBERSORT-ABS | 0.108945 | 0.015838 |
| microenvironment score_XCELL | -0.18449 | 3.98E-05 |
| Monocyte_QUANTISEQ | -0.12014 | 0.007761 |
| Monocyte_XCELL | 0.14329 | 0.001472 |
| Myeloid dendritic cell_MCPCOUNTER | -0.19952 | 8.59E-06 |
| Neutrophil_QUANTISEQ | 0.112313 | 0.012858 |
| Neutrophil_TIMER | -0.11003 | 0.014815 |
| NK cell activated_CIBERSORT-ABS | -0.16817 | 0.000184 |
| NK cell resting_CIBERSORT | 0.164915 | 0.000246 |
| NK cell resting_CIBERSORT-ABS | 0.144062 | 0.001386 |
| NK cell_MCPCOUNTER | -0.20207 | 6.54E-06 |
| NK cell_QUANTISEQ | -0.1849 | 3.82E-05 |
| Plasmacytoid dendritic cell_XCELL | -0.09714 | 0.031566 |
| T cell CD4+ (non-regulatory)_QUANTISEQ | 0.246377 | 3.29E-08 |
| T cell CD4+ effector memory_XCELL | 0.131569 | 0.003527 |
| T cell CD4+ naive_CIBERSORT | 0.104308 | 0.020923 |
| T cell CD4+ naive_CIBERSORT-ABS | 0.104096 | 0.021186 |
| T cell CD4+ naive_XCELL | -0.1531 | 0.000673 |
| T cell CD4+_TIMER | -0.21051 | 2.59E-06 |
| T cell CD8+ central memory_XCELL | -0.19139 | 2.00E-05 |
| T cell CD8+ effector memory_XCELL | -0.09384 | 0.037841 |
| T cell CD8+_CIBERSORT | -0.21997 | 8.78E-07 |
| T cell CD8+_CIBERSORT-ABS | -0.26436 | 2.80E-09 |
| T cell CD8+_EPIC | -0.22896 | 3.00E-07 |
| T cell CD8+_MCPCOUNTER | -0.22865 | 3.12E-07 |
| T cell CD8+_QUANTISEQ | -0.20777 | 3.52E-06 |
| T cell CD8+_TIMER | -0.08935 | 0.04807 |
| T cell CD8+_XCELL | -0.19013 | 2.27E-05 |
| T cell follicular helper_CIBERSORT | -0.28752 | 8.82E-11 |
| T cell follicular helper_CIBERSORT-ABS | -0.36118 | 1.52E-16 |
| T cell gamma delta_XCELL | -0.17976 | 6.29E-05 |
| T cell NK_XCELL | 0.139089 | 0.002029 |
| T cell regulatory (Tregs)_CIBERSORT | -0.11779 | 0.009057 |
| T cell regulatory (Tregs)_CIBERSORT-ABS | -0.18313 | 4.54E-05 |
| T cell regulatory (Tregs)_QUANTISEQ | -0.21209 | 2.17E-06 |
| T cell_MCPCOUNTER | -0.22263 | 6.42E-07 |


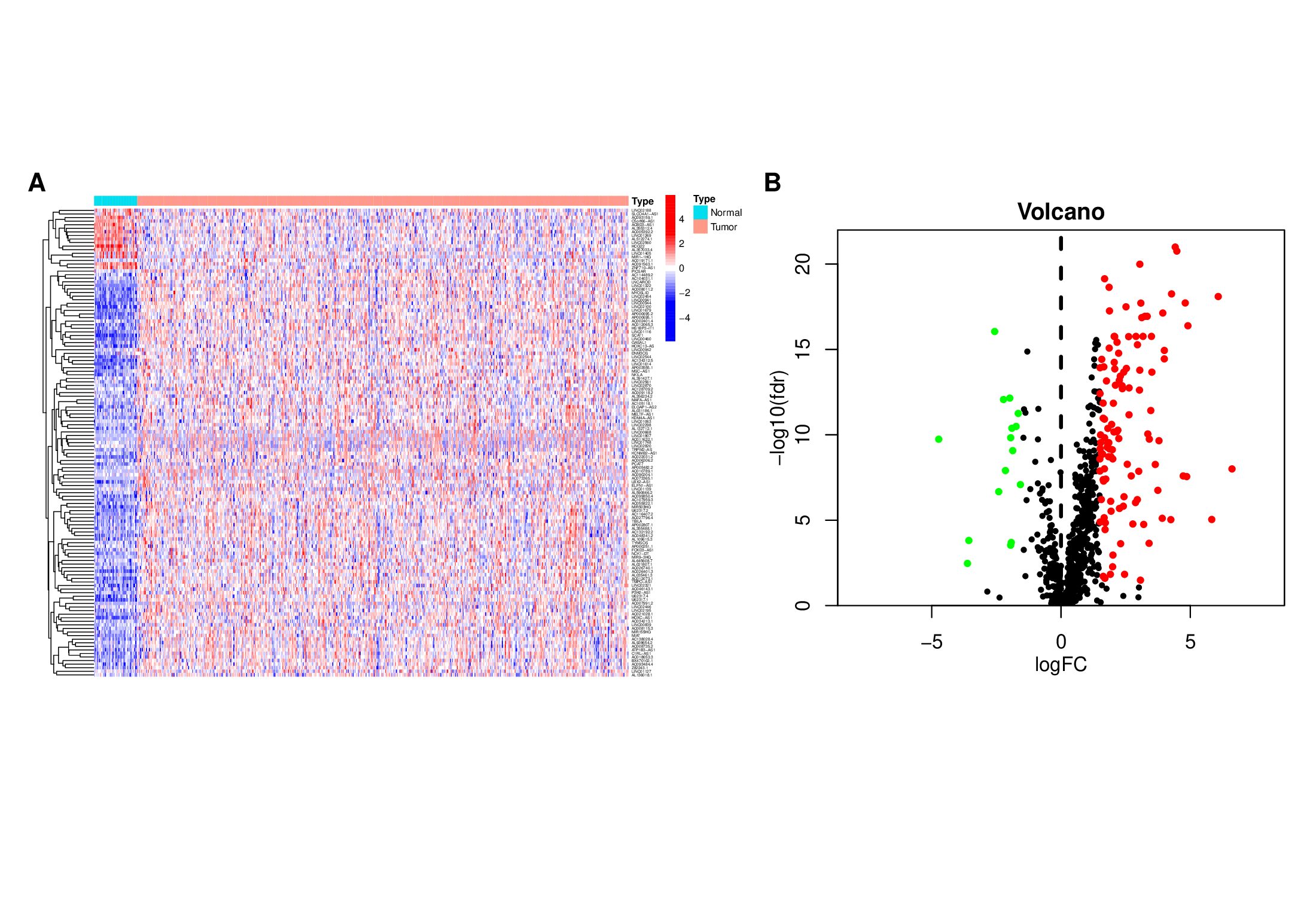


**Figure S1 Heatmap and volcano plot of differently expressed immune-related lncRNAs. (A) Heatmap. (B) Volcano plot**


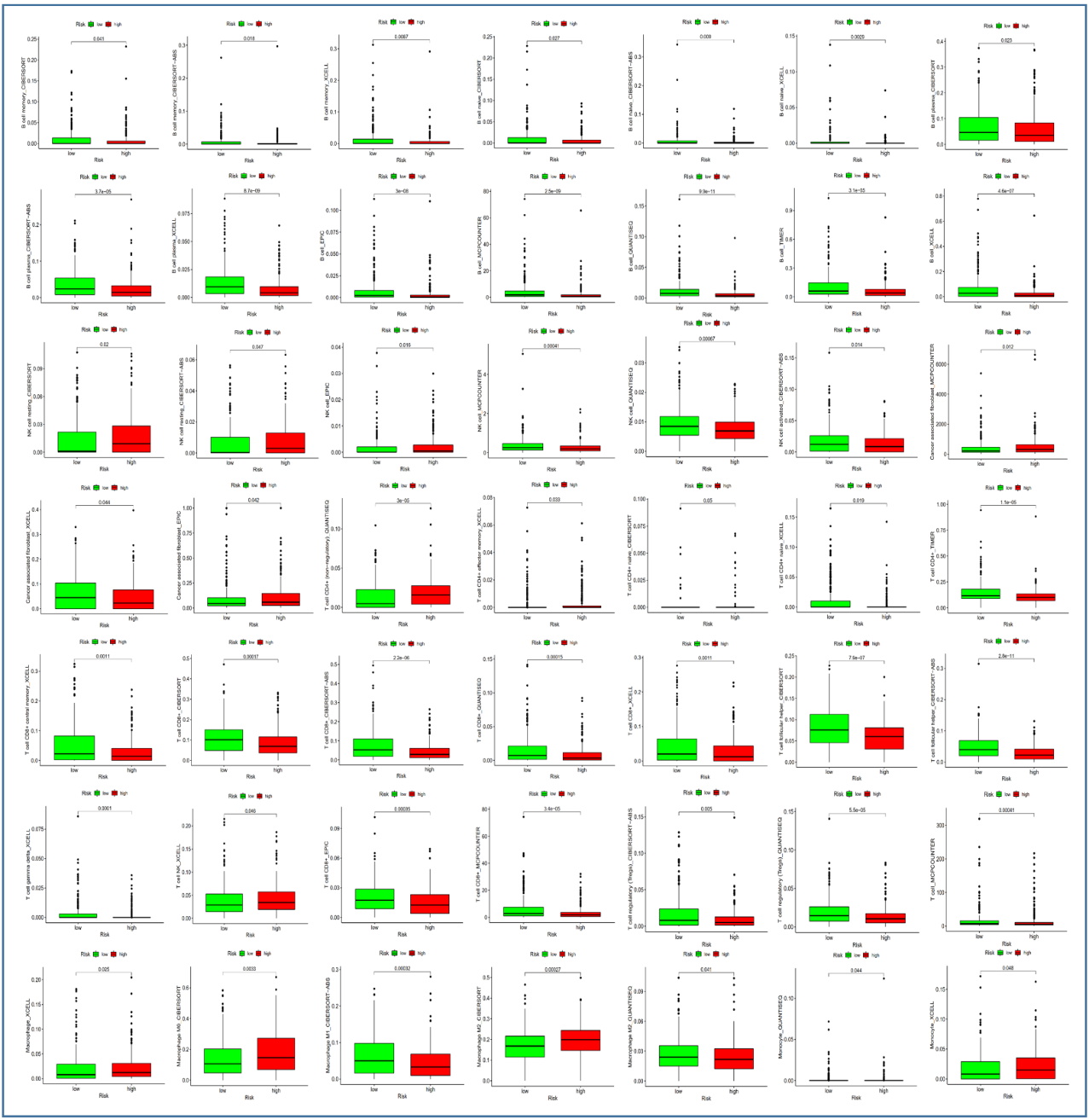


**Figure S2. The representative results of the evaluation of tumor infiltrating immune cells with** **risk assessment model.**
